# Soluble CD14 in cerebrospinal fluid is associated with markers of inflammation and axonal damage in untreated HIV-infected patients: a retrospective cross-sectional study

**DOI:** 10.1186/s12879-016-1510-6

**Published:** 2016-04-21

**Authors:** Sofie Jespersen, Karin Kæreby Pedersen, Birgitta Anesten, Henrik Zetterberg, Dietmar Fuchs, Magnus Gisslén, Lars Hagberg, Marius Trøseid, Susanne Dam Nielsen

**Affiliations:** Department of Infectious Diseases, Viro-immunology Research Unit, Rigshospitalet, University of Copenhagen, Blegdamsvej 9, DK 2100 Copenhagen Ø, Denmark; Department of Infectious Diseases, Institute of Biomedicine, Sahlgrenska Academy, University of Gothenburg, Gothenburg, Sweden; Clinical Neurochemistry Laboratory, Institute of Neuroscience and Physiology, Sahlgrenska Academy, University of Gothenburg, Mölndal, Sweden; Department of Molecular Neuroscience, UCL Institute of Neurology, Queen Square, London, UK; Division of Biological Chemistry, Biocenter, Innsbruck Medical University, Innsbruck, Austria; Department of Infectious Diseases, Oslo University Hospital, Oslo, Norway

**Keywords:** HIV, CNS inflammation, Microbial translocation, sCD14, NFL, Neopterin

## Abstract

**Background:**

HIV-associated cognitive impairment has declined since the introduction of combination antiretroviral treatment (cART). However, milder forms of cognitive impairment persist. Inflammation in the cerebrospinal fluid (CSF) has been associated with cognitive impairment, and CSF neurofilament light chain protein (NFL) and CSF neopterin concentrations are increased in those patients. Microbial translocation in HIV infection has been suggested to contribute to chronic inflammation, and lipopolysaccharide (LPS) and soluble CD14 (sCD14) are markers of microbial translocation and the resulting monocyte activation, respectively. We hypothesised that microbial translocation contributes to inflammation and axonal damage in the central nervous system (CNS) in untreated HIV infection.

**Methods:**

We analyzed paired samples of plasma and CSF from 62 HIV-infected, untreated patients without cognitive symptoms from Sahlgrenska University Hospital, Gothenburg, Sweden. Measurements of neopterin and NFL in CSF were available from previous studies. Plasma and CSF sCD14 was measured using ELISA (R&D, Minneapolis, MN), and plasma and CSF LPS was measured using LAL colorimetric assay (Lonza, Walkersville, MD, USA). Univariate and multivariate regression analyses were performed.

**Results:**

LPS in plasma was associated with plasma sCD14 (*r* = 0.31, *P* = 0.015), and plasma sCD14 was associated with CSF sCD14 (*r* = 0.32, *P* = 0.012). Furthermore, CSF sCD14 was associated with NFL (*r* = 0.32, *P* = 0.031) and neopterin (*r* = 0.32, *P* = 0.012) in CSF. LPS was not detectable in CSF. In a multivariate regression model CSF sCD14 remained associated with NFL and neopterin after adjusting for age, CD4+ cell count, and HIV RNA in CSF.

**Conclusions:**

In a group of untreated, HIV-infected patients LPS was associated with sCD14 in plasma, and plasma sCD14 was associated CSF sCD14. CSF sCD14 were associated with markers of CNS inflammation and axonal damage. This suggest that microbial translocation might be a driver of systemic and CNS inflammation. However, LPS was not detectable in the CSF, and since sCD14 is a marker of monocyte activation sCD14 may be increased due to other causes than microbial translocation. Further studies regarding cognitive impairment and biomarkers are warranted to fully understand causality.

## Background

Prior to introduction of combination antiretroviral treatment (cART) a large proportion of HIV-infected patients developed severe neurocognitive disorders [1, 2]. With the introduction of cART, the incidence of severe HIV-associated neurocognitive disorders decreased, while milder types persists [3, 4].

A number of studies suggest that cognitive impairment may be caused by inflammation in the central nervous system (CNS) [5–8]. Thus, biomarkers of inflammation in the cerebrospinal fluid (CSF) have been associated with cognitive impairment. One such biomarker is neopterin, a marker of monocyte activation which has been found to be elevated in the CSF of HIV-infected patients. The concentration of neopterin decreases after initiation of cART, although it does not reach the concentrations observed in HIV-uninfected persons [9, 10], hence indicating persistent CNS inflammation [11]. Neurofilament light chain protein (NFL) is a marker of CNS axonal damage that has been associated with CNS inflammation. NFL is increased in HIV-infected patients with cognitive impairment or opportunistic CNS infections [12–17].

Systemic inflammation in HIV-infected patients has been reported repeatedly [18–20] and is believed to be partly caused by microbial translocation. Microbial translocation and immune activation are predictors of disease progression and non-AIDS-related morbidity in HIV infection [21–23]. During acute HIV infection, the gastrointestinal mucosa is depleted of T cells leading to translocation of microbial products such as lipopolysaccharide (LPS) into the systemic circulation [24–28]. LPS activates cells of the innate immune system by binding to Toll-like receptor-4 (TLR-4). CD14 needs to be present in order for LPS to activate the receptor. CD14 is expressed on monocytes, macrophages, and neutrophils, and upon stimulation by LPS, as well as other microbial products, CD14 is secreted and cleaved from the cells as soluble CD14 (sCD14) [29].

We hypothesised that microbial translocation contributes to inflammation in the CNS of HIV-infected patients. We therefore aimed to investigate if markers of microbial translocation (LPS) and monocyte activation (sCD14) were detectable in CSF and to determine possible associations between these markers and markers of inflammation and axonal damage in the CNS (NFL, neopterin) in untreated HIV-infected patients.

## Methods

### Participants

Since 1985, HIV-infected patients in Gothenburg, Sweden have been included in a longitudinal study that includes serial sampling of CSF, plasma, and serum. This cohort has previously been described in detail [14]. Lumbar punctures are performed in a standardized manner at least annually and more frequently in connection with antiretroviral treatment initiation or change in treatment. CSF and blood samples are stored at −80 °C in batches. Both asymptomatic and symptomatic patients are included, and as of December 2014 the cohort included 494 patients who had undergone 1805 lumbar punctures. Patients were clinical assessed for neurocognitive function (reporting no challenges in regard to daily living or work).

From this cohort 20 untreated HIV-infected patients without CNS opportunistic infection but with pleocytosis the CSF (>5 cells/μL) indicating HIV-induced inflammation was included in a pilot study in order to determine if it was possible to detect sCD14 and LPS in CSF during HIV infection. The 20 participants from the pilot study did not participate in the following analyses.

Afterwards, 62 untreated, HIV-infected patients without cognitive symptoms or CNS opportunistic infections with samples collected between 2004 and 2014 were selected for further analyses. Untreated patients were selected to avoid confounding effects of cART on microbial translocation and inflammation. 30 patients had pleocytosis.

In order to determine if CD4+ cell count affected either microbial translocation or monocyte activation, patients were selected to have either CD4+ cell count >350 cells/μL (*n* = 31, CD4+ cell count range 380–1400, median 530 cells/μL) or CD4+ cell count <200 cells/μL (*n* = 31, CD4+ cell count range 0–190, median 100 cells/μL). Groups were matched on gender, age, and CSF leucocyte count. The selection was done randomly from the list of patients fulfilling the inclusion criteria (Table 1). Five patients with low CD4+ cell count were treated with trimetoprim/sulfametoxazole including four as prophylaxis against *Pneumocystis jirovecii*, and one patient was treated for *Pneumocystis* pneumonia. Furthermore, one patient was treated for tuberculosis.

**Table 1:** Characteristics of the study population

Untreated HIV-infected patients	
Characteristics	Total sample (*n* = 62)
Age (years, range)	39 (24–68)
Gender (male, *n*(%))	32 (52)
CD4+ cell count (cells/μL)	190 (98–530)
HIV RNA blood (copies/mL)	39587 (8799–172541)
HIV RNA CSF (copies/mL)	4690 (749–28150)
Leucocytes CSF (cells/μL)	5 (1–9)
Serum albumin (g/L)	39 (36–42)
CSF albumin (mg/L)	165 (136–243)
Albumin ratio	3.5 (4.1–5.9)
Mode of transmission (*n* (%)):	
Heterosexual	49 (79)
MSM	11 (18)
IV drug users	2 (3)
AIDS defining diseases (*n* (%)):	
*Pneumocystis jirovecii* pneumonia	1 (2)
Wasting syndrome	2 (3)
Tuberculosis	1 (2)
Biomarkers	
LPS plasma (pg/mL)	102 (78–121)
sCD14 plasma (ng/mL)	2382 (1941–3111)
sCD14 CSF (ng/mL)	222 (161–373)
Neopterin plasma (nmol/L)	12 (9–23)
Neopterin CSF (nmol/L)	15 (8–22)
NFL CSF (ng/L)	410 (250–635)

Data is given as median (IQR), unless otherwise stated

*LPS* lipopolysaccharide, *NFL* neurofilament light, *CSF* cerebrospinal fluid, *MSM* men who have sex with men, *IV* intravenous

The study was approved by the Regional Ethical Review Board in Gothenburg (Ö 588–01) and was performed in accordance with the Helsinki Declaration. All blood and CSF samples were obtained after informed consent of subjects under the IRB-approved protocol.

### Markers of axonal damage, inflammation, and HIV-related factors

CSF NFL was measured using a sensitive sandwich enzyme-linked immunosorbent assay (ELISA) (NF-light® ELISA kit, UmanDiagnostics AB, Umeå, Sweden) as previously described [12, 30]. CSF neopterin was measured using a commercially available radioimmunoassay (Henningtest Neopterin, BRAHMS, Berlin, Germany) [15]. HIV-1 RNA in CSF and plasma were quantified with Roche Amplicor Monitor assay version 1.5 (Hoffman La-Roche, Basel, Switzerland) with a detection limit of 20 copies/mL. Furthermore, routine assessments included CSF white blood cell count and peripheral blood CD4+ cell count as previous described [31].

Valid measurement of neopterin in plasma and CSF, NFL and leucocyte count in CSF were available in 60, 61, 46, and 60 patients, respectively.

### Markers of microbial translocation and monocyte activation

LPS in plasma and CSF was analysed by the Limulus amebocyte lysate colorimetric assay (Lonza, Walkersville, MD) according to the manufacturer’s instructions and with the following modifications: Samples were diluted 10-fold to avoid interference with background colour, and preheated to 68 °C for 12 min prior to analysis to dissolve immune complexes, as previously described [32, 33].

Soluble CD14 in plasma and CSF was analysed using ELISA according to the manufacturer’s instructions (R&D, Minneapolis, MN).

### Statistics

Data are given as median (interquartile range (IQR)). A *P* value ≤0.05 was considered significant. When comparing groups, the Student *T*-test and Mann Whitney *U*-test was used as appropriate. Uni- and multivariate linear regression models were used to assess associations and adjusted for possible confounders. When appropriate data were log-transformed (ln). In order to investigate possible associations between microbial translocation and inflammation in the CSF, multivariate linear regression models were performed with NFL and neopterin as the dependent variables, and sCD14 in the CSF was added as an independent variable. The model was adjusted for age, HIV RNA in the CSF, and CD4+ cell count. Since patients were included in two CD4+ cell count groups, CD4+ cell count was added to the model as both a continuous variable and as a nominal variable. Only the continuous variable was significant and therefore used in the final model. The analyses were performed using IBM SPSS 22.0 (Statistics for Windows, IBM Corp., Armonk, NY).

## Results

### Characteristics of the study population

The pilot study included 20 untreated, asymptomatic, HIV-infected patients with CSF pleocytosis as a sign of HIV-induced inflammation. Both LPS and sCD14 was detectable in all plasma samples. However, LPS was not detectable in any CSF samples whereas CSF-sCD14 was detectable in all CSF samples (data not shown). Hence, only sCD14 was measured in CSF in the subsequent analyses.

Samples from 62 untreated, HIV-infected patients without clinical evidence of impaired cognitive function and with CD4+ cell count either above 350 or below 200 cells/μL were selected for further analysis. Characteristics of the study population are shown in Table 1.

### Detection of LPS in plasma and sCD14 in plasma and CSF

Valid measurement of LPS in plasma and sCD14 in plasma was obtained in all 62 participants whereas sCD14 in CSF was obtained in 61 participants.

Patients with plasma sCD14 above median had higher plasma LPS compared to patients with sCD14 below median (91 pg/mL vs. 117 pg/mL, *P* = 0.042), whereas there were no differences in age, gender, CD4+ cell count, or plasma HIV RNA between these two groups. However, when dividing the patients into two groups with sCD14 in CSF above or below median, no differences were found in regard to age, gender, CD4+ cell count, plasma HIV RNA, plasma LPS, or CFS HIV RNA (all *P* values >0.05).

### Soluble CD14 and immune deficiency

Increased concentrations of sCD14 in plasma was found in patients with CD4+ cell count <200 compared to patients with CD4+ cell count >350 (median of 2579 ng/mL (IQR 2061–3641) vs. 2357 ng/mL (IQR 1174–2959) (*P* = 0.031)). Furthermore, concentration of sCD14 in CSF was 308 (IQR 196–385) in the group with CD4+ cell count <200 vs. 201 (IQR 90–317) in the group with CD4+ cell count >350 (*P* = 0.015). Leucocyte cell counts in CSF were median 3 cells/μL (IQR 1–11) and 5 cells/μL (IQR 4–9) (*P* = 0.406) in the group with CD4+ cell count <200 vs. the group with CD4+ cell count >350. Concentrations of CSF sCD14 was associated with CSF leucocyte cell count in a univariate analysis (*r* = 0.73, *P* = 0.049).

### Markers of microbial translocation associated with inflammation in the CNS

**Fig. 1:**
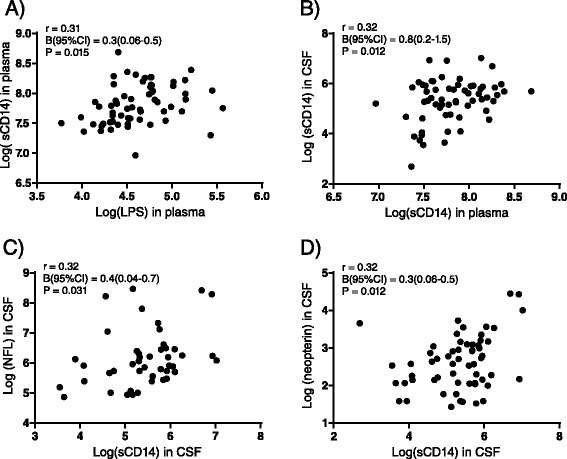
Univariate analyses. Markers of microbial translocation (LPS), monocyte activation (sCD14), axonal damage (NFL), and CNS inflammation (neopterin) were measured in paired samples of cerebrospinal fluid (CSF) and plasma from 62 untreated HIV-infected patients. Univariate analysis were performed after Log(ln) transformation due to skewed data. (**a**) plasma LPS and plasma sCD14, (**b**) plasma sCD14 and CSF sCD14, (**c**) CSF sCD14 and CSF NFL, (**d**) CSF sCD14 and CSF neopterin. CSF = cerebrospinal fluid, LPS = lipopolysaccharide, NFL = neurofilament light chain, sCD14 = soluble CD14

Univariate analyses demonstrated associations between plasma LPS and plasma sCD14 (*r* = 0.31, *P* = 0.015), as well as borderline significant association with CSF sCD14 (*r* = 0.24, *P* = 0.059). Furthermore, plasma sCD14 was associated with CSF sCD14 (*r* = 0.32, *P* = 0.012, Fig. 1) and tended to correlate with plasma neopterin (*r* = 0.24, *P* = 0.060). Importantly, CSF sCD14 was associated with a higher concentration of both NFL (*r* = 0.32, *P* = 0.031) and neopterin in the CSF (*r* = 0.32, *P* = 0.012, Fig. 1).

In a multivariate linear regression model, CSF sCD14 remained associated with NFL (*P* = 0.039) and neopterin (*P* = 0.002) when adjusted for age, CD4+ cell count, and HIV RNA in CSF. In line with previous studies, higher age (*P* = 0.001) and lower CD4+ cell count (*P* = 0.001) were associated with higher NFL in multivariable analysis [12]. Higher HIV RNA in CSF (*P* < 0.001) and lower CD4+ cell count (*P* < 0.001) were associated with higher neopterin. When LPS was added to the model, no association was found with NFL or neopterin.

## Discussion

In this study of untreated, HIV-infected patients, sCD14, a marker of microbial translocation and monocyte activation was detectable in both plasma and CSF. High CSF and plasma sCD14 was not explained by higher HIV RNA or lower CD4+ cell counts, while a significant association between plasma sCD14 and LPS was found. Furthermore, significant associations between CSF sCD14 and markers of inflammation and axonal damage in the CSF were found, independent of age, HIV RNA, and CD4+ cell count. Hence, it is possible that elevated monocyte activation, partly driven by microbial translocation, may contribute to the pathogenesis in CNS by promoting inflammation.

Infection of the CNS occurs early during HIV infection [34, 35]. HIV enters the CNS unassisted or in infected monocytes that cross the blood–brain barrier (BBB) [36–38]. In the CNS, HIV and migrated immune cells lead to the production of pro-inflammatory cytokines and further immune activation. Inflammation in the CNS creates a neurotoxic environment resulting in damage of the neurons [39]. Increased NFL reflects axonal damage [40], and neopterin is a marker of activated monocytes that is used to assess inflammation in the CNS in HIV-infected patients. In pre-cART studies high concentration of HIV RNA in the CSF was associated with severity of cognitive impairment [37, 38]. However, the association between HIV RNA and cognitive impairment in the cART era has not been as strong or consistent, indicating that other factors may also play a role in the development of HIV-associated cognitive impairment [38, 41–43].

Immune activation and inflammation is a hallmark of HIV infection and is caused by several factors including HIV replication, ART toxicity, co-infections, loss of regulatory cells, and microbial translocation [44]. Depletion of immune cells around the gastrointestinal tract leads to increased permeability for microbial products like LPS [45]. LPS is a part of the Gram negative bacterial wall and is frequently used in studies as a marker of microbial translocation. LPS cannot penetrate a normal functioning BBB, however, inflammation caused by LPS may make the BBB more permeable for HIV and activated monocytes [46, 47]. To our knowledge LPS measurements in the CSF have not previously been reported.

The binding of LPS to TLR-4 results in secretion of pro-inflammatory cytokines and sCD14 from activated monocytes. We found significant associations between plasma LPS and plasma sCD14 as well as borderline significant association with CSF sCD14. Activated monocytes that have migrated to the CNS is the most likely source of sCD14 in the CSF, as sCD14 has not been reported to be produced from residing monocytes (microglia and astrocytes) [48]. Neopterin, which also is a marker of monocyte activation, is on the other hand produced by residing monocytes in the CSF [49]. This makes sCD14 an interesting biomarker in the CSF, as it potentially reflects the level of monocyte migration into the CNS supported by a positive correlation between CSF cell count and CSF sCD14. However, CD14 has been shown to possess several other functions besides binding LPS. CD14 also acts as a co-receptor for several other TLRs and microbial products which may explain the lack of consistency in studies investigating LPS in association with CD14 [50, 51].

Several studies have shown that markers of microbial translocation are associated with cognitive impairment in HIV-infected patients [7, 8, 52–54]. Thus, CNS HIV Anti-Retroviral Therapy Effects Research (CHARTER) and The National NeuroAIDS Tissue Consortium (NNTC) groups have shown that LPS and sCD14 in plasma and in the CSF were increased in patients with impaired cognitive function [7, 8, 53]. Furthermore, increased sCD14 persists even after effective cART [55]. We found strong associations between CSF sCD14 and NFL, even after adjustment for age, HIV RNA, and CD4+ cell count, indicating that activated monocytes may contribute to axonal damage in the CNS, albeit this cross sectional study does not allow us to determine causality. Furthermore, our findings are supported by a recent study of CSF sCD14 and NFL in untreated HIV-infected patients [17].

Guidelines to assess cognitive function are based on the use of neuropsychological test [56]. Studies have shown that even in well treated HIV-infected individuals on cART, as many as 50 % have been reported to have neurocognitive disorders [57–59]. However, neuropsychological tests are time consuming, require specialised staff, and are not available in all clinical settings. Furthermore, neuropsychological tests are not able to differentiate between active or residual cognitive impairment [60]. It has been suggested that a combination of biomarkers could be used to assess CNS inflammation and guide in diagnosing HIV-associated CNS damage [61]. sCD14 could be a potential candidate as it has been established that activated monocytes play an important role in the pathogenesis of cognitive impairment in HIV infection [7, 8, 52–54], however in the present study markers of CNS inflammation and axonal damage was associated with CSF sCD14, but not with plasma sCD14. The present study did not include neurocognitive testing and only included naïve HIV-infected individuals. Data on neurocognitive performance as well as individuals on cART could add valuable information in future studies.

This study was limited by limited sample size and the cross-sectional design. Furthermore, measuring microbial translocation using LPS is challenging since the method is sensitive to both time and contamination [62]. However, this method is well established in our laboratory with an inter assay coefficient of variation < 10 %. Also, other microbial products besides LPS, including peptidoglycan, Pam_3_CSK_4_, polyI:C, and CpG DNA, cross the gastrointestinal mucosa and may activate the immune system and drive inflammation [50, 51]. Recently it was found that two types of sCD14 exits [63]. A differential measurement of these may have provided additional information. Furthermore, neuropsychological evaluation would have supplied valuable information.

## Conclusion

Significant associations between sCD14 in CSF and markers of CNS inflammation and axonal damage were found suggesting that monocyte activation, possibly driven by microbial translocation, may contribute to CNS inflammation. Further investigations into the pathogenesis of cognitive impairment in HIV infection, as well as potential biomarkers are needed, in order to increase the understanding and supply reliable methods of diagnosing these conditions.

### Ethics approval and consent to participate

The study was approved by the Regional Ethical Review Board in Gothenburg (Ö 588–01) and was performed in accordance with the Helsinki Declaration. All blood and CSF samples were obtained after informed consent of subjects under the IRB-approved protocol.

### Consent for publication

Not applicable.

### Availability of data and materials

All data are available upon request.

## Abbreviations

AIDS: acquired immunodeficiency syndrome
BBB: blood brain barrier
cART: combination antiretroviral treatment
CD: cluster of differentiation
CHARTER: CNS HIV Anti-Retroviral Therapy Effects Research
CNS: central nervous system
CSF: cerebrospinal fluid
ELISA: enzyme-linked immunosorbent assay
HIV: human immunodeficiency virus
IQR: interquartile range
IV: intravenous
LAL: limulus amebocyte lysate
LPS: lipopolysaccharide
mL: millilitre
mm3: cubic millimetre
n: subsample size
NFL: neurofilament light chain protein
ng: nano gram
nmol: nano mole
NNTC: the National NeuroAIDS Tissue Consortium
Pg: Pico gram
RNA: ribonucleic acid
sCD14: soluble CD14
TLR: toll- like receptors
